# Comprehensive Analysis Reveals Potential Molecular Targets in Juvenile Dermatomyositis

**DOI:** 10.1155/bri/1147461

**Published:** 2026-03-06

**Authors:** Chunyan Chen, Haifa Qiao

**Affiliations:** ^1^ Institute for Chinese Medicine Frontier Interdisciplinary Science and Technology, Shaanxi University of Chinese Medicine, Xixian New Area, Xianyang, Shaanxi, 712046, China, sntcm.edu.cn; ^2^ College of Acupuncture-Moxibustion and Tuina, Shaanxi University of Chinese Medicine, Xixian New Area, Xianyang, Shaanxi, 712046, China, sntcm.edu.cn; ^3^ Xianyang Key Laboratory of Neurobiology (Acupuncture), Shaanxi University of Chinese Medicine, Xixian New Area, Xianyang, Shaanxi, 712046, China, sntcm.edu.cn

**Keywords:** differential gene expression analysis, juvenile dermatomyositis, miRNA, protein–protein interaction network, transcription factors, WGCNA

## Abstract

Juvenile dermatomyositis (JDM) is a rare autoimmune disease primarily affecting children, characterized by muscle weakness and skin lesions. This study identifies 145 genes significantly associated with JDM through differential gene expression analysis, weighted gene coexpression network analysis (WGCNA), protein–protein interaction network analysis, and miRNA and transcription factor (TF) prediction, using blood and muscle microarray sequencing datasets. Functional enrichment analysis indicates that these genes are involved in crucial biological processes, including cytokine‐mediated signaling, extracellular matrix organization, and immune response. Further analysis reveals key TFs (*e.g.,* STAT1 and NFKB1) and miRNAs (*e.g.,* hsa‐miR‐127‐3p and hsa‐miR‐17‐5p) that may regulate the expression of these critical genes in JDM. The findings provide new insights into the molecular mechanisms of JDM and offer potential targets for future diagnostic and therapeutic strategies.

**Summary**
 This research addresses juvenile dermatomyositis (JDM), a rare autoimmune disease affecting children, by identifying 145 genes linked to its development. The study analyzed blood and muscle samples, revealing important genes involved in immune response and inflammation. Key findings highlight specific transcription factors and miRNAs that may regulate these genes, offering new insights into the disease’s underlying mechanisms. Understanding these molecular details is crucial, as it opens up potential pathways for developing targeted diagnostic tools and therapies, ultimately improving outcomes for children suffering from JDM. This work is a significant step toward enhancing our approach to this challenging condition.

## 1. Introduction

Juvenile dermatomyositis (JDM) is a rare autoimmune disease that primarily affects children, characterized by muscle weakness and skin lesions [1, 2]. Although the exact etiology of JDM remains unclear, immune system abnormalities are believed to play a crucial role in its pathogenesis [3, 4]. Recent advances in gene expression profiling techniques have enabled researchers to explore genes and regulatory mechanisms associated with JDM in depth, providing new clues for disease diagnosis and treatment [1, 2]. Previous studies have shown that cytokine‐mediated signaling, extracellular matrix organization, and immune response play key roles in the pathogenesis of JDM [3–5]. However, research on the molecular mechanisms of JDM remains limited, necessitating further systematic analyses to identify key genes and regulatory molecules associated with the disease.

**Figure 1:**
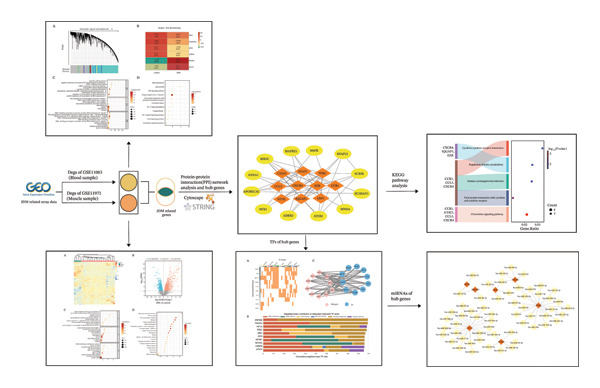
Overview of the workflow of the study.

In this study, we conducted a comprehensive analysis of the GSE11083 and GSE11971 datasets to identify genes significantly differentially expressed in blood and muscle samples from JDM patients (Figure 1). Using weighted gene coexpression network analysis (WGCNA), we identified gene modules closely related to JDM pathogenesis and performed functional enrichment analysis to reveal the key biological processes (BP) involving these genes. Additionally, we used transcription factor (TF) prediction and miRNA analysis to identify key TFs and miRNAs regulating these critical genes. The results provide new insights into the molecular mechanisms of JDM and potential targets for future diagnostic and therapeutic strategies.

## 2. Materials and Methods

### 2.1. Differential Gene Expression Analysis

We retrieved high‐throughput sequencing data related to JDM from the GEO database and selected two relevant microarray datasets. The first dataset, GSE11083 [6], consists of array sequencing data from peripheral blood mononuclear cells (PBMC), including 14 JDM samples and 13 normal control samples. The second dataset, GSE11971 [7], includes array sequencing data from skeletal muscle, containing 19 JDM samples and 4 control samples. Expression matrix data for each dataset were downloaded and normalized. Differential gene expression was calculated using the Limma package. For GSE11083, we used the limma package for differential expression gene (DEG) detection. Given the relatively balanced sample size and the subtle gene expression changes typical in PBMC, we applied a relaxed cutoff (*p* *-*value < 0.05) to enhance sensitivity. Here, the *p*‐values were calculated based on moderated *t*‐statistics provided by the eBayes function in limma, which improves variance estimation in small‐sample settings. This is more lenient than the original publication’s thresholds (*p* *-*value < 0.001 and |log2FC| > 0.85) [6], aiming to retain more candidate genes for downstream co‐expression and network analysis. For GSE11971, we adopted more stringent criteria (*q-*value < 0.01) to ensure specificity, given the small sample size and strong transcriptomic signals in skeletal muscle tissues. False discovery rate (FDR) correction was performed using the Benjamini–Hochberg method to reduce false positives. This strategy is in line with recent analysis pipelines using this dataset [8].

### 2.2. WGCNA Analysis

WGCNA [9] was performed on the GSE11083 dataset to identify gene modules significantly associated with JDM. Genes with an average expression level below 1 and a coefficient of variation (variance/mean) less than 0.3 were filtered out. The remaining genes were subjected to hierarchical clustering to detect sample outliers. Specifically, we performed hierarchical clustering on all samples and generated a sample dendrogram (Figure S1). Two samples, GSM279791 and GSM279787, were found to deviate significantly from the main cluster. To reduce potential artifacts caused by outliers in the network analysis, we set the cut height to 75 based on the clustering tree and excluded these two samples.

An optimal soft‐threshold power of 4 was selected based on the criterion R^2 > 0.9 (Figure S2). Gene modular clustering was performed with parameters set to deepSplit = 4, cutHeight = 0.9, and minModuleSize = 100. The correlation between each gene module and phenotype was calculated, and genes from modules significantly associated with JDM were selected for subsequent gene functional enrichment analysis.

To assess the robustness of identified modules, we implemented a bootstrap‐based resampling procedure [10–12]. Specifically, we repeated the WGCNA analysis 100 times using 96% randomly sampled individuals in each iteration. The stability of each gene was quantified as the proportion of runs in which it was assigned to the same module. After comparing the results under various stability score thresholds, a cutoff of 0.6 was chosen as it provided a balance between gene retention and module consistency. To evaluate the robustness of module detection under different network construction parameters, we additionally performed WGCNA using an alternative set of clustering parameters (softPower = 4, cutHeight = 0.9, minModuleSize = 150, deepSplit = 4). We then compared the functional consistency of JDM‐associated modules across the two parameter settings.

### 2.3. Gene Functional Enrichment Analysis

Gene Ontology (GO) and KEGG functional enrichment analyses were conducted on the target gene sets to determine their significantly related biological functions and signaling pathways. The DAVID tool [13] was used for GO functional enrichment analysis, covering significant enrichments in BP, cellular components (CC), and molecular functions (MF). KEGG pathway enrichment analysis was also performed to identify significant signaling pathways associated with JDM.

### 2.4. Protein–Protein Interaction (PPI) Network Construction and Analysis

The STRING database was used to construct the PPI network of JDM‐related genes, with the minimum required interaction score set to > 0.4. The PPI network was visualized using Cytoscape 3.10.1 [14] software, and hub genes were identified using the cytoHubba [15] plugin. Nine algorithms (MCC, MNC, Degree, EPC, EcCentricity, Closeness, Radiality, Betweenness, Stress) were integrated to select the top 10 hub genes as key genes related to JDM.

### 2.5. TTF and miRNA Network Analysis

The ChEA3 [16] database was used to query TFs of the 10 hub genes and construct the TF‐gene network to analyze their roles in the pathogenesis of JDM. For miRNA analysis, the miRabel [17] database was used to identify miRNAs targeting the 10 hub genes. The miRabel database incorporates multiple databases and experimental validation data, including PITA [18], miRanda [19], SVMicrO [20], TargetScan [21], and Experimental verification (ExpVal). miRNAs recorded in at least five databases and experimental evidence were selected to construct the miRNA‐gene network and analyze their regulatory roles in JDM. The interaction network was visualized using Cytoscape.

## 3. Results

### 3.1. JDM‐Related Genes and Biological Functions in Muscle Tissue

In the muscle tissue dataset (GSE11971), we conducted DEG analysis and functional enrichment analysis. Using the Limma method, we identified 1506 DEGs (Table S1), with 878 genes significantly upregulated and 628 genes significantly downregulated in JDM samples. Heatmap analysis (Figure 2(a)) demonstrated that the differences in gene expression clearly separated the control and JDM groups, indicating the importance of these genes in distinguishing JDM samples. The volcano plot (Figure 2(b)) further illustrated the distribution of these DEGs, highlighting the number and significance of upregulated and downregulated genes.

Figure 2Differentially expressed genes and functional enrichment analysis in muscle tissue (GSE11971) related to JDM. (a) Gene expression heatmap. (b) Volcano plot of differentially expressed genes. The volcano plot shows the distribution of differentially expressed genes in the GSE11971 dataset. The red dots represent upregulated genes, blue dots represent downregulated genes, and gray dots represent nonsignificant genes. (c) GO enrichment analysis bubble chart of differentially expressed genes. This bubble chart displays the top 10 GO enrichment analysis results. The enriched GO terms are categorized into three main classes: biological process (BP), cellular component (CC), and molecular function (MF). Bubble size represents the number of enriched genes, while color indicates the enrichment significance (*p* *-*value). (d) KEGG enrichment analysis bubble chart of differentially expressed genes. This bubble chart shows the top 20 KEGG pathway enrichment analysis results. Bubble size represents the number of enriched genes, while color indicates the enrichment significance (*p* *-*value).

**Figure:**
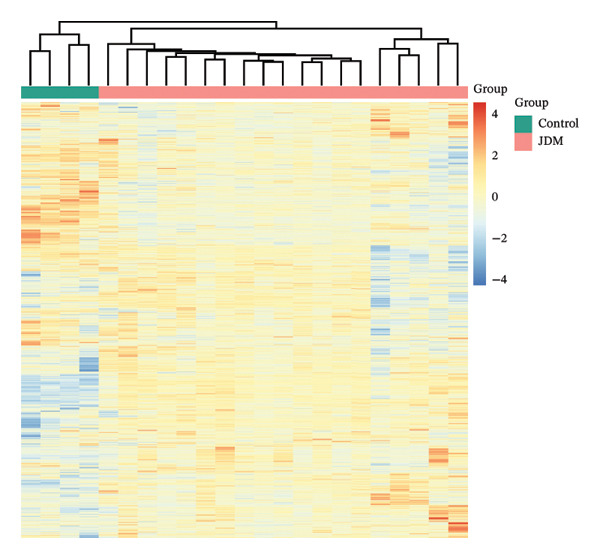
(a)

**Figure:**
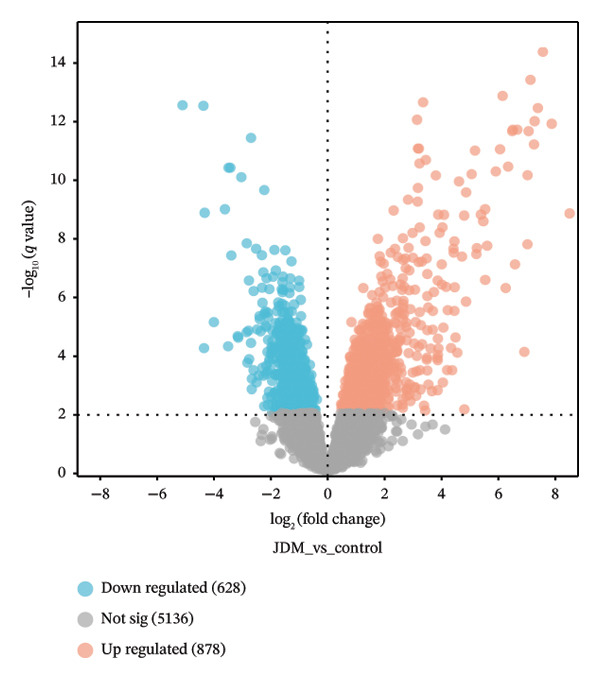
(b)

**Figure:**
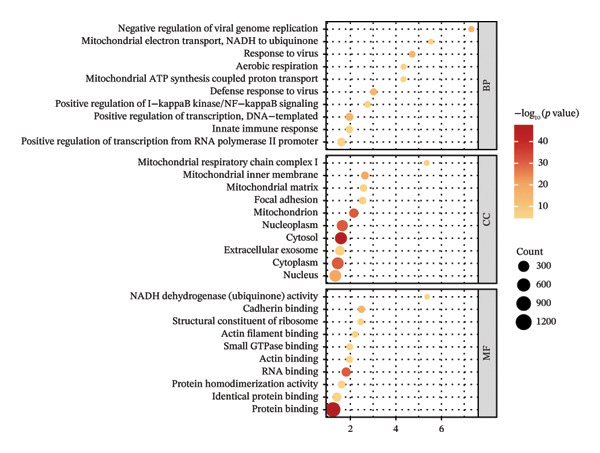
(c)

**Figure:**
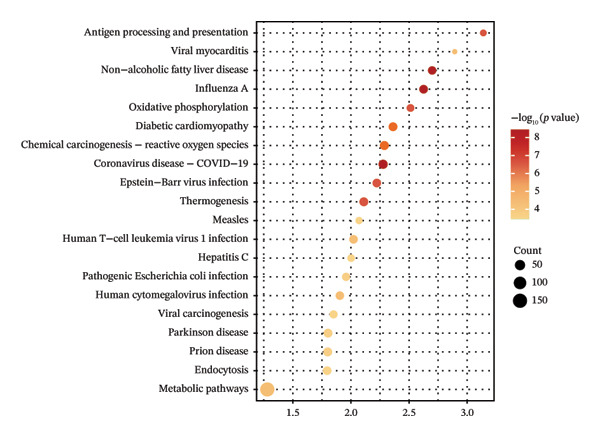
(d)

We performed GO and KEGG pathway enrichment analyses on these DEGs (Table S2). The GO enrichment bubble chart (Figure 2(c)) revealed that DEGs were significantly enriched in BP such as response to viruses, negative regulation of viral genome replication, defense response, mitochondrial electron transport, positive regulation of I‐kappaB kinase/NF‐kappaB signaling, positive regulation of transcription from DNA template, aerobic respiration, innate immune response, and mitochondrial ATP synthesis coupled proton transport. The KEGG pathway enrichment bubble chart (Figure 2(d)) showed significant enrichment of these DEGs in several pathways, including influenza A, nonalcoholic fatty liver disease, coronavirus disease (COVID‐19), diabetic cardiomyopathy, chemical carcinogenesis–reactive oxygen species, Epstein–Barr virus infection, antigen processing and presentation, thermogenesis, oxidative phosphorylation, and human T‐cell leukemia virus 1 infection. These results provide crucial insights into understanding the pathophysiological mechanisms of JDM.

### 3.2. JDM‐Related Genes and Functional Enrichment Analysis in Blood Samples

We used WGCNA to analyze blood samples (GSE11083) to identify gene expression modules significantly associated with JDM. A gene co‐expression network was constructed with a soft threshold of 4 (Figure S2). Modular clustering analysis generated six gene modules (Figure S3, Table S3), each represented by different (Figure 3(a)).

Figure 3Gene modules and functional enrichment analysis significantly associated with JDM in blood samples (GSE11083). (a) Gene clustering tree and module color map from WGCNA analysis. Genes were clustered into different modules based on expression patterns, with each module represented by a different color. (b) Module–trait relationship chart. Darker colors indicate stronger correlations, with red representing positive correlations and green representing negative correlations. The numbers indicate the correlation coefficient and the significance (*p* *-*value) in parentheses. (c) GO analysis of genes in the brown module significantly associated with JDM: this chart shows the top 10 significantly enriched biological processes (BP), cellular components (CC), and molecular functions (MF). Colors represent −log_10_ (*p* *-*value), and the size of the dots indicates the number of genes. (d) KEGG pathway analysis of genes in the brown module significantly associated with JDM: this chart shows significantly enriched pathways (*p* *-*value < 0.05). Colors represent −log_10_ (*p* *-*value), and the size of the dots indicates the number of genes.

**Figure:**
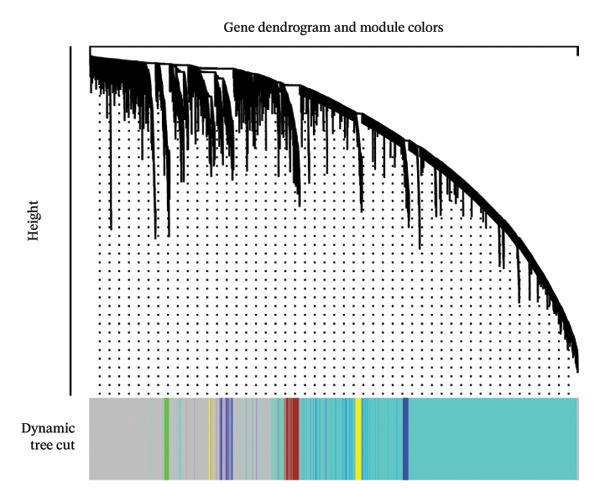
(a)

**Figure:**
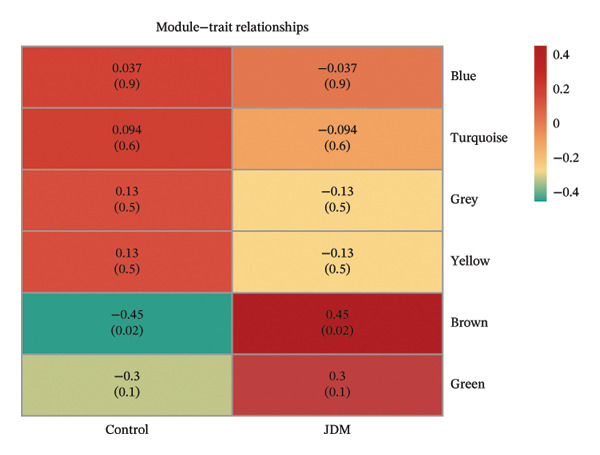
(b)

**Figure:**
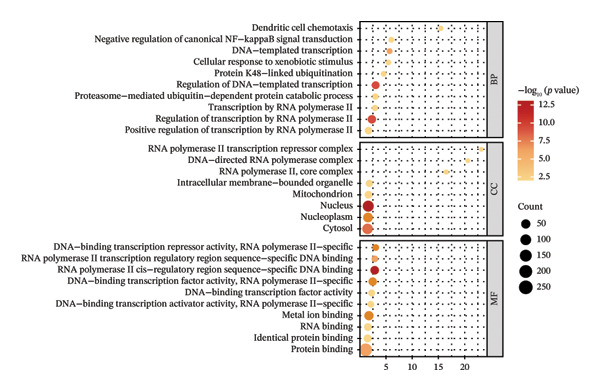
(c)

**Figure:**
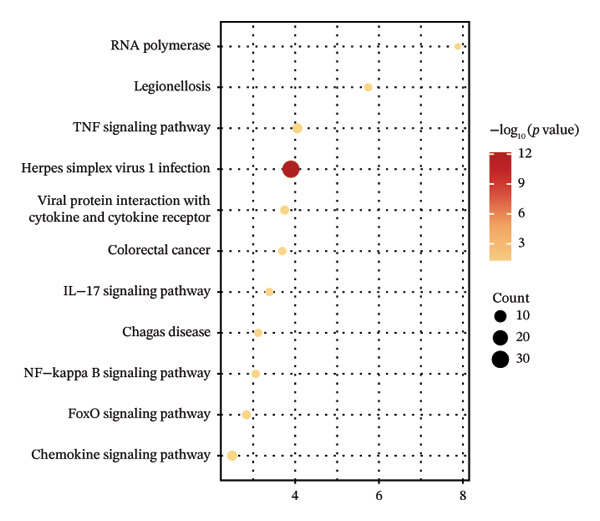
(d)

We further calculated the correlation between each gene module and JDM as well as control groups. Figure 3(b) shows the correlation between each module and JDM, with the brown module exhibiting a significant positive correlation with JDM (correlation coefficient = 0.45, *p* *-*value = 0.02) and a significant negative correlation with the control group (correlation coefficient = −0.45, *p* *-*value = 0.02). Functional enrichment analysis was performed on genes in the brown module significantly associated with JDM (Table S4). GO analysis results showed that these genes were significantly enriched in various BP, CC, and MF, such as dendritic cell chemotaxis, protein K48‐linked ubiquitination, and transcription by RNA polymerase II (Figure 3(c)). KEGG pathway analysis indicated that these genes were significantly enriched in several signaling pathways, including the TNF signaling pathway, Herpes simplex virus 1 infection, and NF‐kappa B signaling pathway (Figure 3(d)).

To assess the stability and biological relevance of the JDM‐associated module identified by WGCNA, we performed 100 bootstrap resampling iterations using the original parameters (softPower = 4, minModuleSize = 100, cutHeight = 0.9, deepSplit = 4), reconstructing the network from 96% of randomly selected samples each time. Gene stability scores were defined as the frequency of assignment to the original module across all iterations. Given the modest sample size (*n* = 26), we compared thresholds of 0.5, 0.6, and 0.7, and selected 0.6 to balance gene retention and stability, resulting in about half of the brown module genes classified as stable core genes (Tables S5 and S6).

In addition, we repeated the bootstrap stability analysis using an alternative set of WGCNA parameters (softPower = 4, minModuleSize = 150, cutHeight = 0.9, deepSplit = 4) (Tables S7 and S8, Figure S4). The resulting JDM‐associated modules were consistent with those identified using the original parameters, with similar functional enrichment profiles (Figure S5, Tables S9 and S10) and substantial overlap of stable core genes (Figure S6). Pathway enrichment analysis of these stable core genes consistently highlighted immune‐related pathways, such as TNF, IL‐17, NOD‐like receptor, NF‐kappa B signaling, and ubiquitin‐mediated proteolysis (Figure S5). These results demonstrate that, despite the limited sample size, the identified modules and stable core genes are not only reproducible across different analytical settings but also enriched in consistent immune‐related pathways, suggesting their biological relevance and stability.

Through these analyses, we identified that genes in the brown module are closely related to the pathogenesis of JDM and identified several potential BP and signaling pathways that provide important clues for further research and therapy. Additionally, differential expression analysis of this sample identified 989 differentially expressed genes (Table S11), with 450 genes upregulated and 539 genes downregulated in JDM (Figure S7). After removing duplicates, 1350 JDM‐related genes were identified in blood samples.

### 3.3. Genes and Functional Analysis Related to JDM in Blood and Muscle

We intersected the 1350 JDM‐related genes obtained from GSE11083 with the DEGs from GSE11971, ultimately identifying 145 genes associated with JDM in both blood and muscle samples (Figure 4(a), Table S12). To better understand the biological functions of these 145 genes in JDM, we conducted GO analysis (Table S13). The results of showed significant enrichment of these genes in various BP, CC, and MF. The top 10 significantly enriched GO terms include cytokine‐mediated signaling pathway, extracellular matrix, and protein binding (Figure 4(b)). These results suggest that these genes may be involved in several critical BP and functions in JDM. Next, we used the STRING database to construct a PPI network for the 145 genes, including 140 edges and 93 nodes, to identify key regulatory genes. The PPI network revealed complex interactions among multiple genes, and the cytoHubba plugin identified the top 10 hub genes (Table S14), including TFRC, STAT1, CXCR4, CD163, CCL5, CCR1, NT5E, EZR, LRP1, and IQGAP1 (Figure 4(c), orange nodes, Table 1, the PPI network of 145 genes is shown in Figure S8). These hub genes may play crucial roles in the pathogenesis of JDM. We performed KEGG pathway analysis on these 10 hub genes to determine the signaling pathways they may be involved in. The results indicated significant enrichment in several important signaling pathways, including cytokine–cytokine receptor interaction, regulation of actin cytoskeleton, human cytomegalovirus infection, chemokine signaling pathway, and viral protein interaction with cytokine and cytokine receptor (Figure 4(d), Table S15). These pathways are closely related to the pathogenesis of JDM, providing potential targets for further research and therapy.

Figure 4Genes and functional enrichment analysis related to JDM in blood and muscle tissue. (a) Intersection of JDM‐related genes from GSE11083 with DEGs from GSE11971, identifying 145 genes associated with JDM in both blood and muscle samples. (b) Gene Ontology (GO) analysis results of the 145 genes, showing the top 10 significantly enriched GO terms (*p* *-*value < 0.05). Categories include biological processes (BP), cellular components (CC), and molecular functions (MF). Colors represent −log_10_ (*p* *-*value), and the size of the dots indicates the number of genes. (c) Subnetwork of the protein–protein interaction (PPI) network constructed from the 145 shared genes, showing only the top 10 hub genes and their first‐order interacting neighbors, based on STRING analysis. Orange nodes represent hub genes. (d) KEGG pathway analysis of the 10 hub genes, showing significantly enriched pathways (*p* *-*value < 0.05). Colors represent −log_10_ (*p-*value), and the size of the dots indicates the number of genes.

**Figure:**
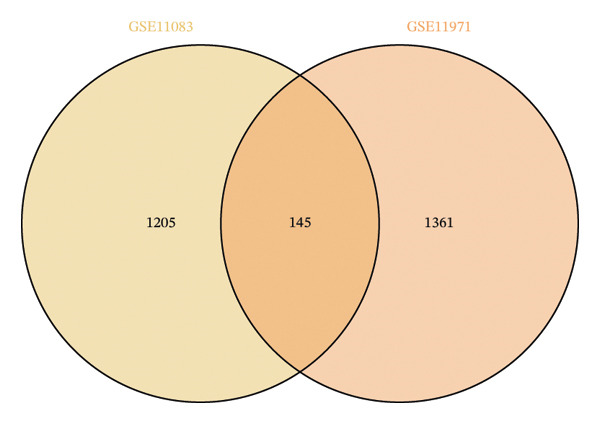
(a)

**Figure:**
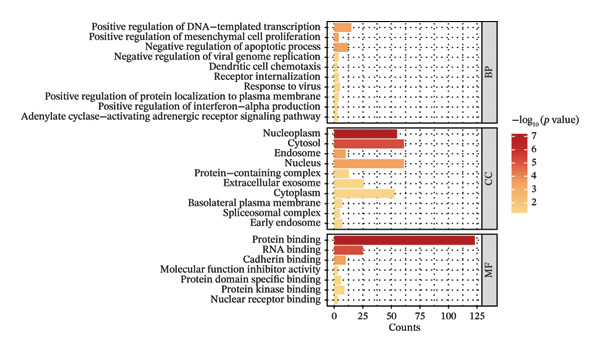
(b)

**Figure:**
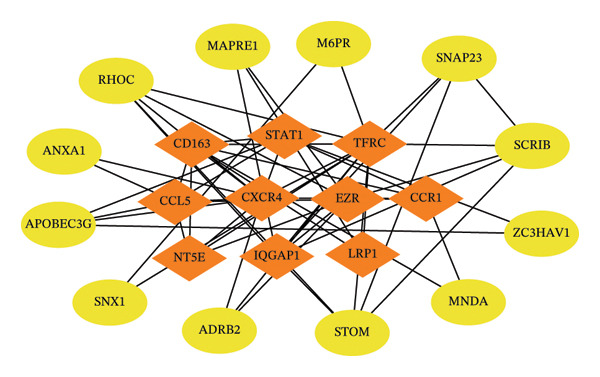
(c)

**Figure:**
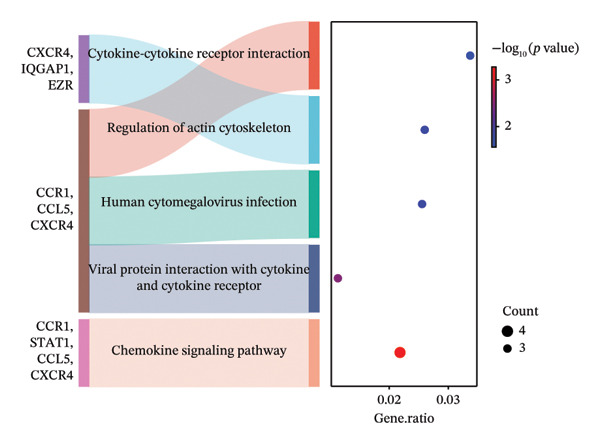
(d)

**Table 1:** Description of 10 hub genes.

Gene name	Degree	Description
TFRC	13	Transferrin Receptor
STAT1	11	Signal Transducer and Activator of Transcription 1
CXCR4	11	C‐X‐C Motif Chemokine Receptor 4
CD163	8	CD163 Molecule
CCL5	8	C‐C Motif Chemokine Ligand5
CCR1	5	C‐C Motif Chemokine Re 1
NT5E	7	5′‐Nucleotidase Ecto (CD73)
EZR	8	Ezrin
LRP1	6	LDL Receptor Related Protein 1
IQGAP1	7	IQ Motif Containing GTPase Activating Protein 1

Through the above analyses, we systematically identified key genes and pathways related to JDM, revealing the potential biological functions and interactions of these genes in JDM, and providing important clues for understanding the pathogenic mechanisms of JDM.

### 3.4. TF and miRNA Regulatory Network Analysis

To identify key TFs regulating the 10 hub genes, we used the ChEA3 database for predictive analysis. First, Figure 5(a) shows the predicted TFs from various data sources, including ARCHS4 Coexpression, ENCODE ChIP‐seq, Enrichr Queries, GTEx Co‐Expression, Literature ChIP‐seq, and ReMap ChIP‐seq. These data sources provide multidimensional evidence supporting the TF predictions. Through comprehensive analysis, the ChEA3 database identified the top 10 TFs significantly involved in regulating the JDM‐related hub genes (Figure 5(b)). These top 10 TFs include STAT1, NFKB1, IRF9, CEBPB, HIF1A, TWIST2, ZNF469, TFEC, MSC, and NCOA3. To further elucidate the interactions between these TFs and hub genes, we constructed an interaction network between the top 10 TFs and the 10 hub genes. Figure 5(c) shows these interactions. This network graph reveals the regulatory relationships between each TF and multiple hub genes.

Figure 5Transcription factor prediction and interaction network of the 10 hub genes. (a) Sources of transcription factors predicted by the ChEA3 database for the 10 hub genes. Various data sources include ARCHS4 coexpression, ENCODE ChIP‐seq, enrichr queries, GTEx coexpression, literature ChIP‐seq, and ReMap ChIP‐seq. (b) Top 10 transcription factors identified by the ChEA3 database. These transcription factors play significant roles in regulating the JDM‐related 10 hub genes. (c) Interaction network of the top 10 transcription factors with the hub genes. Blue circles represent transcription factors, pink diamonds represent hub genes, and edges indicate predicted regulatory interactions.

**Figure:**
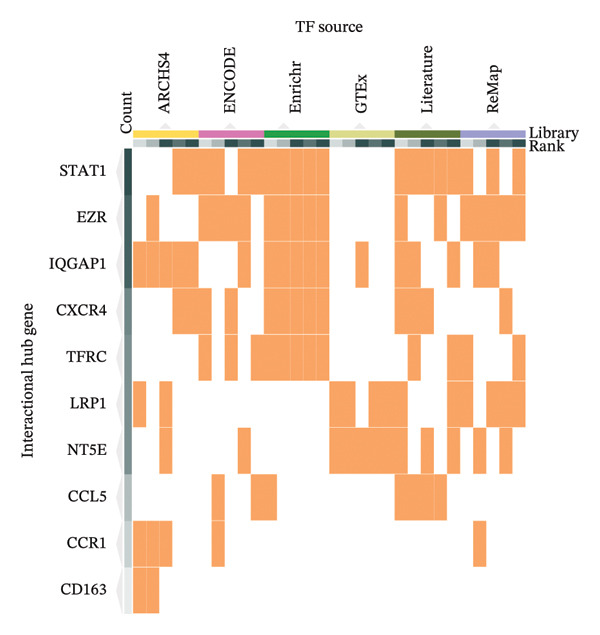
(a)

**Figure:**
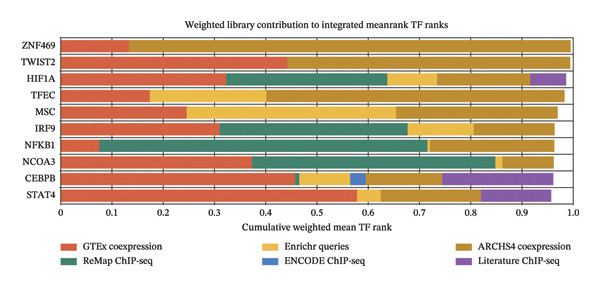
(b)

**Figure:**
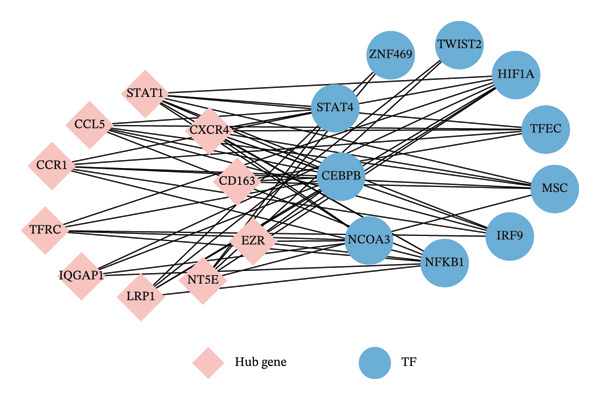
(c)

**Figure 6:**
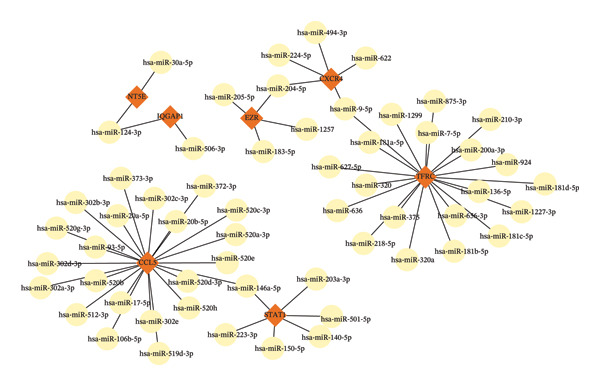
miRNAs–interactional hub genes interaction network. Red diamond nodes represent hub genes, yellow circular nodes represent miRNAs, and edges represent interaction relationships.

To identify key miRNAs regulating the 10 hub genes, we used the miRabel database for predictive analysis (Table S16). The results showed that 57 miRNAs have significant regulatory relationships with 7 hub genes (Figure 6). These miRNAs may play crucial roles in the JDM by regulating the expression of hub genes. For example, CCL5 and TFRC have significant interactions with multiple miRNAs, such as hsa‐miR‐17‐5p and hsa‐miR‐93‐5p, suggesting that these miRNAs may influence the pathological process of JDM by regulating CCL5 and TFRC.

Through the TF and miRNA prediction and network analysis mentioned above, we systematically identified TFs and miRNAs regulating the 10 hub genes. These TFs and miRNAs may play important roles in the pathogenesis of JDM through complex gene regulatory networks. These findings provide important clues for further research on the TF and miRNA regulatory networks in JDM and potential therapeutic targets.

## 4. Discussion

Through a comprehensive analysis of the GSE11083 and GSE11971 datasets, we identified 145 genes significantly associated with JDM. GSE11083 (blood) and GSE11971 (muscle) represent transcriptomic profiles from different tissues of JDM patients. Due to inherent tissue‐specific gene expression, the DEGs identified from each dataset showed limited overlap (145 genes, ∼9.6% (145/1406)). This is expected in multi‐tissue analyses and reflects distinct regulatory mechanisms involved in JDM pathogenesis. Despite the modest overlap, these shared genes demonstrated consistent differential expression across tissues, suggesting their potential as core regulators and meaningful biomarkers. Functional enrichment analysis of these genes revealed their involvement in several critical BP, including cytokine‐mediated signaling, extracellular matrix organization, and immune response. These processes likely play key roles in the pathophysiology of JDM. Specifically, the brown module identified by WGCNA analysis is closely related to JDM onset, and its genes may serve as biomarkers or therapeutic targets.

Our findings are consistent with previous reports. Cytokine signaling, extracellular matrix changes, and immune responses are widely reported in JDM and other autoimmune diseases [22–25], supporting our enrichment results. However, some enriched terms—particularly those related to viral infections such as COVID‐19—may reflect overlapping mechanisms of systemic inflammation or dataset‐specific artifacts, rather than JDM‐specific biology. Therefore, while these results provide a valuable reference for hypothesis generation, they require careful interpretation and subsequent experimental validation.

Using the STRING database and the cytoHubba plugin, we identified the top 10 hub genes (TFRC, STAT1, CXCR4, CD163, CCL5, CCR1, NT5E, EZR, LRP1, IQGAP1). STAT1 is a well‐established regulator of immune and interferon‐related responses [26–28]. CXCR4 and CCL5 are also important in immune cell migration and inflammatory responses [29, 30]. Among them, TFRC (iron uptake, immune cell proliferation) [31, 32] and EZR (cell morphology and migration) [31, 32] show potential immunological relevance. These genes remain hypothetical candidates in JDM and require experimental validation.

Using ChEA3, we identified the top 10 TFs, including STAT1, NFKB1, and IRF9. These TFs play crucial roles in immune response, inflammatory processes, and cell survival and apoptosis. STAT1 and NFKB1, in particular, have been extensively studied in autoimmune diseases [26–28, 33]. TF prediction was based on integrated mean rank values from multiple data sources, including co‐expression, ChIP‐seq, and literature. Among the top TFs, HIF1A, IRF9, NFKB1, NCOA3, CEBPB, and STAT4 had at least one ChIP‐seq–supported interaction. No JDM‐specific validation has yet been reported, highlighting the need for future multi‐level experimental confirmation.

Recent single‐cell transcriptomic studies of JDM [34–39] have highlighted type I interferon pathway activation in immune cells and inflammatory responses in structural cells (e.g., CD4^+^/CD8^+^ T cells, NK cells, B cells) and inflammatory responses in structural cells (e.g., smooth muscle cells, fibroblasts). Our hub genes (e.g., STAT1, CXCR4) did not fully overlap with single‐cell results, likely due to differences in sample source, resolution, and analytical approach. Nevertheless, there is convergence at the pathway level, such as interferon signaling, inflammatory response, and immune cell migration, suggesting mechanistic complementarity. In addition, comparison with bulk RNA‐seq studies (e.g., GSE3307) [40] showed partial reproducibility, with 13 overlapping DEGs, supporting stability of our findings. Together, our results uncover core pathways associated with JDM and provide a complementary perspective to existing single‐cell and microarray studies.

miRNAs also play a significant role in gene expression regulation. Using miRabel, we predicted 57 miRNAs regulating 7 hub genes. For example, hsa‐miR‐127‐3p and hsa‐miR‐17‐5p may regulate STAT1 and CXCR4, and hsa‐miR‐127‐3p has roles in autoimmune diseases [41, 42]. A previous study constructed an miRNA–mRNA network based on muscle‐specific DEGs [43]. However, none of the predicted miRNA–target pairs have been experimentally validated in JDM. These predicted TFs and miRNAs provide a valuable foundation for future studies.

Although this study systematically identified key genes and molecular networks related to JDM, some limitations remain. First, the identified TFs and miRNAs were predicted computationally without IDM‐specific validation. In future, we plan focused validation experiments, including immunohistochemical analysis of STAT1 and CD163 in JDM muscle biopsies, to verify their roles. Second, this study is based on public datasets with limited sample size and cross‐sectional design. Given the heterogeneity of JDM, future work should involve larger cohorts, longitudinal sampling, and multi‐tissue datasets, complemented by single‐cell or spatial transcriptomic to refine cell‐type‐specific mechanisms. Moreover, future studies integrating multiomics data and multicenter clinical cohorts will be essential to further validate and extend the robustness of these findings.

Overall, this study provides new insights into JDM molecular mechanisms and potential targets. With further experimental research, these findings may translate into clinical applications for improved diagnosis and therapy.

## 5. Conclusions

This study systematically identified key genes, TFs, and miRNAs significantly associated with JDM by comprehensively analyzing blood and muscle samples from JDM patients. Specific conclusions include: Analysis of the GSE11083 and GSE11971 datasets identified 145 genes significantly differentially expressed in JDM. These genes are enriched in critical BP such as cytokine‐mediated signaling, extracellular matrix organization, and immune response, indicating their important roles in JDM pathology; WGCNA analysis identified the brown module closely related to JDM onset, with genes in this module potentially serving as specific biomarkers or therapeutic targets for JDM, offering new directions for diagnosis and treatment; Predictive analysis using the ChEA3 database identified several key TFs, including STAT1, NFKB1, and IRF9. The interaction network between TFs and hub genes highlights their critical roles in regulating gene expression related to JDM, further supporting their significance in JDM; multiple miRNAs predicted by the miRabel database, such as hsa‐miR‐124‐3p and hsa‐miR‐17‐5p, may play roles in JDM by regulating key genes. These miRNAs also have important roles in other autoimmune diseases, further validating their potential regulatory functions in JDM.

In summary, this study provides new insights into the molecular mechanisms of JDM and potential targets for future therapeutic strategies. With further experimental research, these findings have the potential to translate into clinical applications, providing better diagnostic and therapeutic methods for JDM patients.

## Author Contributions

C.C. and H.Q. conceived of the article and revised the manuscript. C.C. analyzed all data and wrote the draft manuscript.

## Funding

This work was supported by the Shaanxi Fundamental Science Research Project for Chemistry & Biology (Grant No. 22JHQ049) and Natural Basic Research Program of Natural Science of Shaanxi Province (Grant No. 2019JM‐399).

## Disclosure

A version of this manuscript was presented as a preprint, titled “Comprehensive analysis reveals potential molecular targets in Juvenile Dermatomyositis,” and is available at https://www.authorea.com/doi/full/10.22541/au.172872207.79522683/v1 [44].

## Conflicts of Interest

The authors declare no conflicts of interest.

## Supporting Information

Fig. S1. GSE11083 sample clustering to select abnormal samples.

Fig. S2. Soft thresholds are selected in WGCNA analysis.

Fig. S3. Clustering of gene modules in WGCNA analysis.

Fig. S4. The module–trait relationship chart of the WGCNA module constructed using the parameters soft threshold = 4, minModuleSize = 150, and deepSplit = 4.

Fig. S5. KEGG pathway analysis of genes in modules significantly associated with JDM of two different WGCNA parameters.

Fig. S6. Intersection of JDM‐related stable core genes from two different WGCNA parameters.

Fig. S7. Volcano map of GSE11083 differential gene analysis.

Fig. S8. Protein–protein interaction (PPI) network of the 145 genes, constructed using the STRING database.

Table S1. GSE11971 differential expression gene results.

Table S2. GO and KEGG analysis of 1506 differentially expressed genes.

Table S3. Module‐related genes for WGCNA analysis of GSE11083.

Table S4. Brown module‐related gene GO KEGG enrichment.

Table S5. The WGCNA analysis used the original parameters (soft threshold = 4, minModuleSize = 100, deepSplit = 4) to calculate the gene stability scores under 100 bootstrap sampling.

Table S6. The WGCNA analysis was conducted using the original parameters (soft threshold = 4, minModuleSize = 100, deepSplit = 4) for 100 bootstrap samplings and the proportion of stable core genes of brown modules under different stability scores.

Table S7. WGCNA analysis: Gene stability scores were calculated using parameters (soft threshold = 4, minModuleSize = 150, deepSplit = 4) after 100 bootstrap samplings.

Table S8. The WGCNA analysis employed parameters (soft threshold = 4, minModuleSize = 150, deepSplit = 4) for 100 bootstrap samplings and the proportion of stable core genes of blue modules under different stability scores.

Table S9. The WGCNA analysis used the original parameters (soft threshold = 4, minModuleSize = 100, deepSplit = 4) to conduct 100 bootstrap sampling for the functional enrichment of the core genes of the brown module stability.

Table S10. The WGCNA analysis employed parameters (soft threshold = 4, minModuleSize = 150, deepSplit = 4) for 100 bootstrap samplings and performed functional enrichment of the stable core genes of blue modules under different stability scores.

Table S11. Results of GSE11083 differentially expressed gene.

Table S12. 145 common JDM‐related genes of GSE11083 and GSE11971.

Table S13. 145 JDM‐related genes GO and KEGG enrichment result.

Table S14. Top 10 hub genes from nine algorithms of cytoHubba.

Table S15. KEGG enrichment result of 10 hub genes.

Table S16. miRNA prediction result of 10 hub genes.

## Supporting information


**Supporting Information** Additional supporting information can be found online in the Supporting Information section.

## Data Availability

Data are openly available in a public repository that does not issue DOIs. The data that support the findings of this study are openly available in the Gene Expression Omnibus (GEO) database at https://www.ncbi.nlm.nih.gov/geo/, reference numbers GSE11083 [6] and GSE11971 [7]. All R scripts used for data preprocessing, differential expression analysis, WGCNA construction, input data, and visualization are available at https://github.com/chenchunyan/JDM-analysis-pipeline. The GitHub repository also contains detailed documentation, including package dependencies, and instructions for reproducing the key results presented in this manuscript.
