# Supplementary material for: Comprehensive Analysis Reveals Potential Molecular Targets in Juvenile Dermatomyositis
Source: Biochem Res Int. 2026 Mar 6;2026:1147461. doi: 10.1155/bri/1147461 (PMC12966348; doi:10.1155/bri/1147461)
Supplement: Supplementary file 1 — Supporting Information Additional supporting information can be found online in the Supporting Information section. [file BRI-2026-1147461-s001.zip › Supplementary_figures.docx]

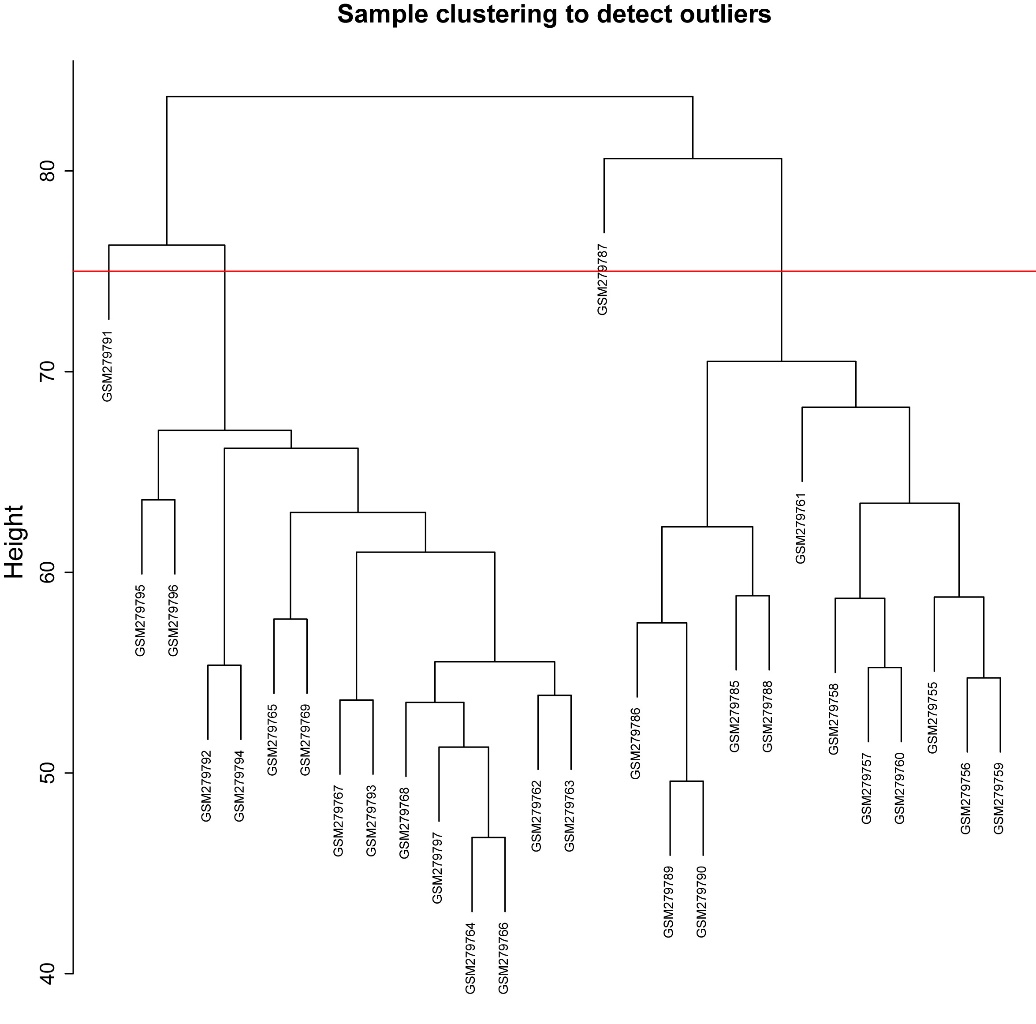


Fig. S1. GSE11083 sample clustering to select abnormal samples.

The red line is the threshold and the sample above the red line is considered an outlier and is filtered out


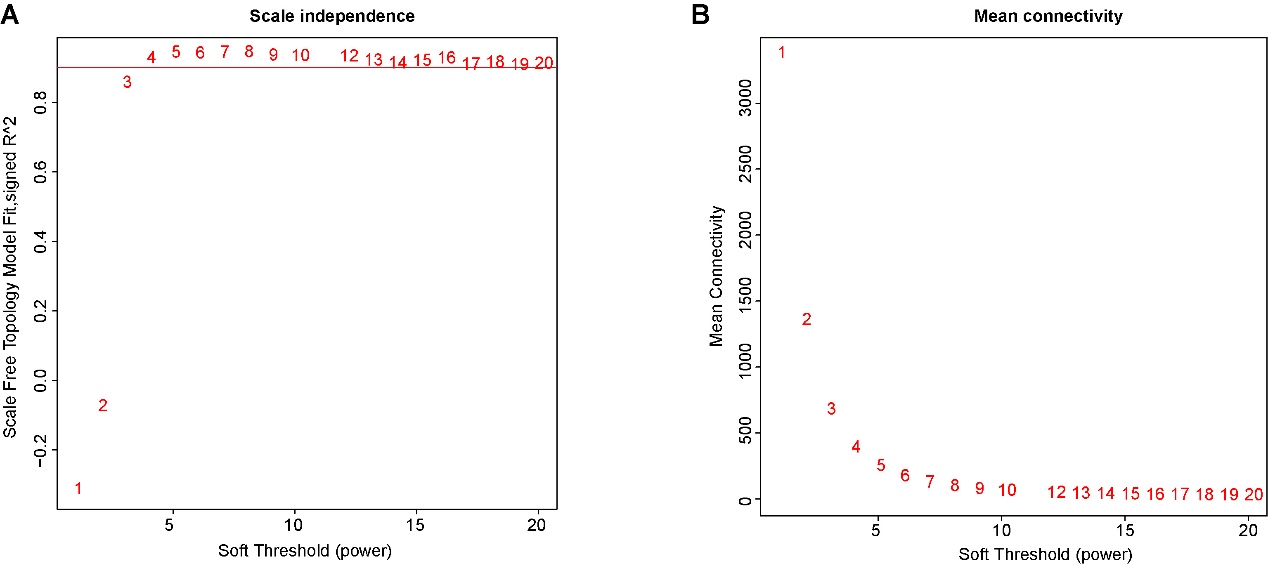


Fig. S2. Soft thresholds are selected in WGCNA analysis.

**A.** Scale independence. This plot displays the scale-free topology model fit represented by R², across different soft threshold powers. As the soft threshold power increases from 1 to 20, the R² value stabilizes around 0.9, indicating a robust scale-free topology The red horizontal line marks the threshold of R² = 0.9, suggesting that the soft threshold value achieves the desired network topology.

B. Mean connectivity. This plot illustrates the mean connectivity of the network as a function of soft threshold power. As the power increases, mean connectivity decreases significantly, indicating that higher soft threshold values lead to sparser networks. This trend helps in identifying an optimal balance between scale-free topology and network sparsity for subsequent network construction.


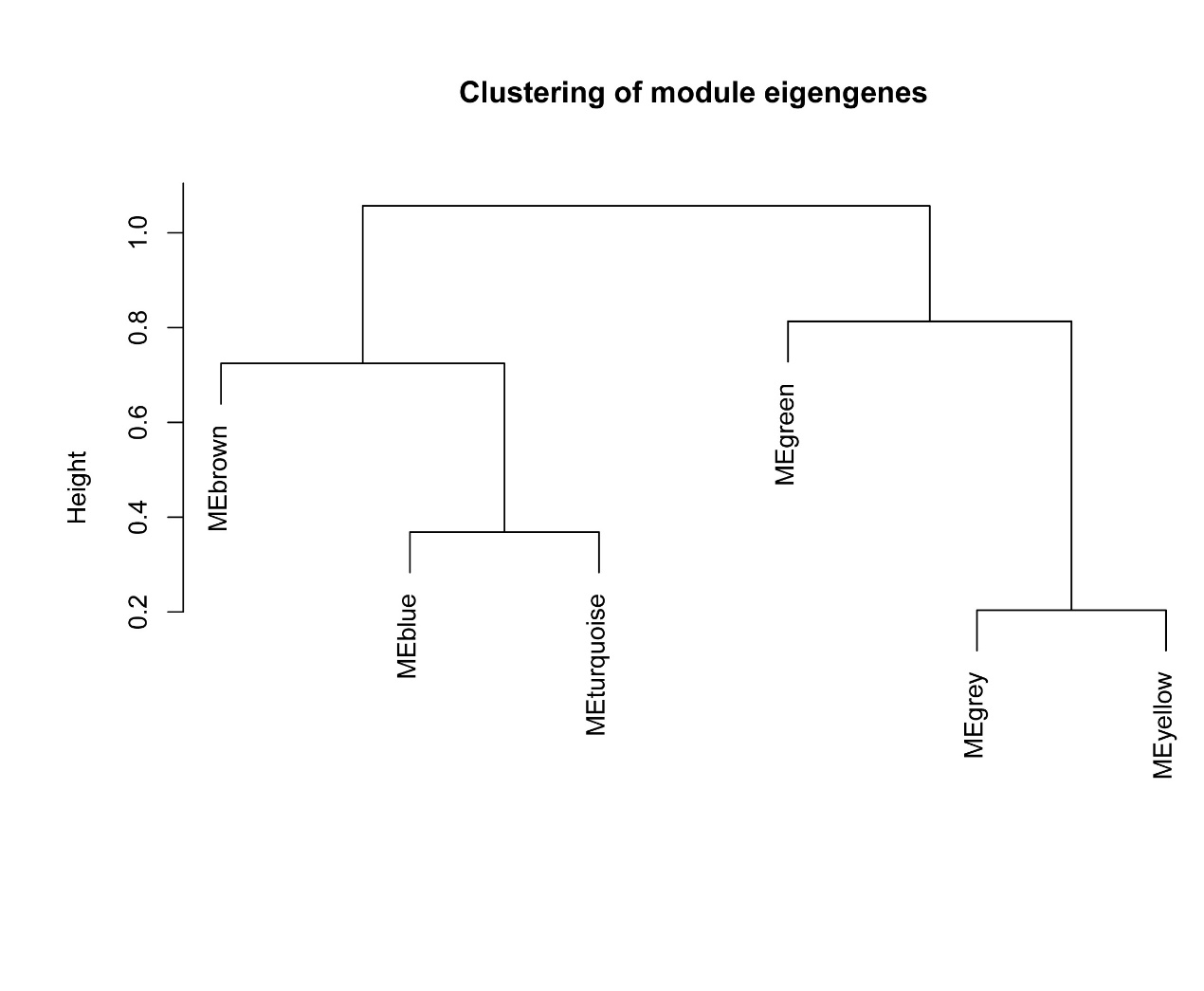


Fig. S3. Clustering of gene modules in WGCNA analysis


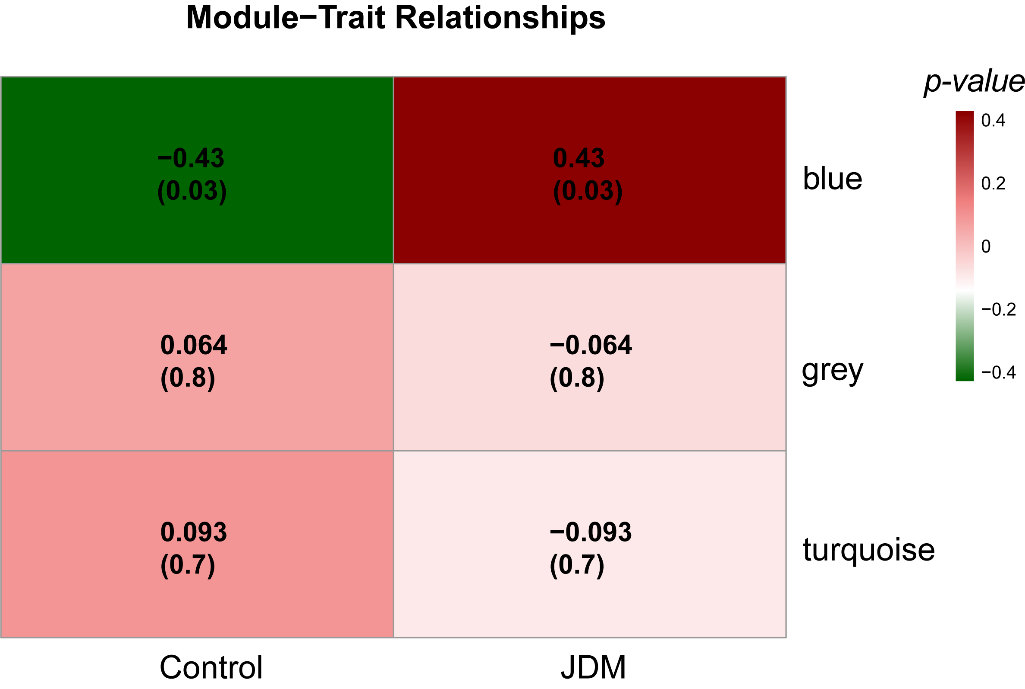


Fig. S4. The Module-trait relationship chart of the WGCNA module constructed using the parameters soft threshold = 4, minModuleSize = 150, and deepSplit = 4. This chart displays the correlation between each gene module and Juvenile Dermatomyositis (JDM) as well as control groups. Darker colors indicate stronger correlations, with dark red representing positive correlations and green representing negative correlations. The numbers indicate the correlation coefficient and the significance (*p-value*) in parentheses.


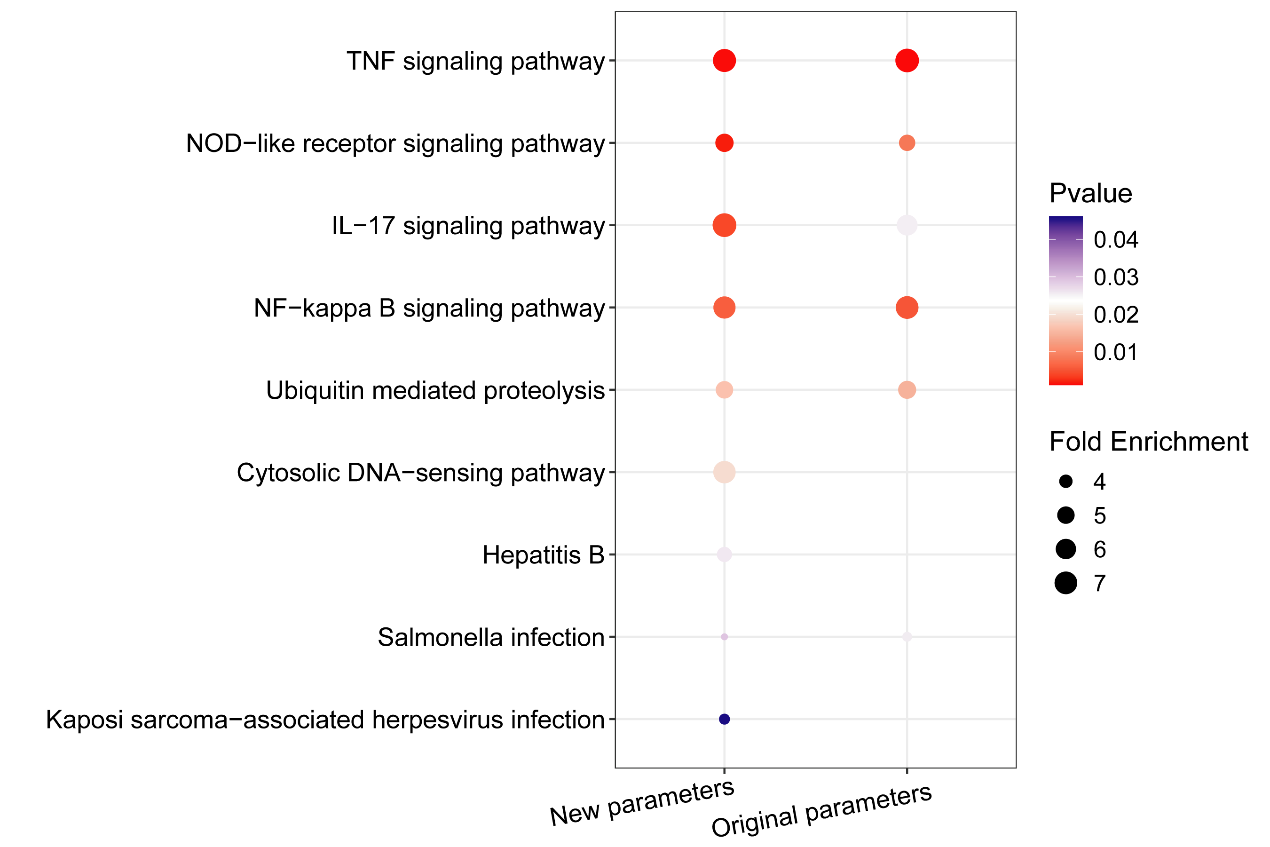


Fig. S5. KEGG pathway analysis of genes in modules significantly associated with JDM of two different WGCNA parameters. Original parameters refer to the original parameters used in the article (soft threshold = 4, minModuleSize = 100, deepSplit = 4), while new parameters represent the more conservative parameters tested (soft threshold = 4, minModuleSize = 150, deepSplit = 4). This chart conducts a KEGG pathway enrichment analysis on the genes with a stability score greater than 0.6, which are constructed under two parameters within the significant correlation module of JDM. This chart shows significantly enriched pathways (*p-value* < 0.05). Colors represent *p-value*, and the size of the dots indicates the fold enrichment of genes.


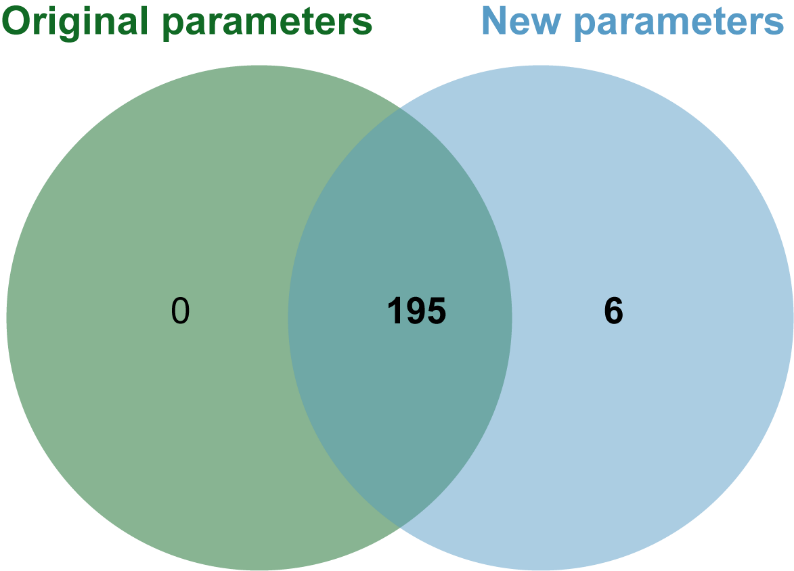


Fig. S6. Intersection of JDM-related stable core genes from two different WGCNA parameters. Original parameters refer to the original parameters used in the article (soft threshold = 4, minModuleSize = 100, deepSplit = 4), while new parameters represent the more conservative parameters tested (soft threshold = 4, minModuleSize = 150, deepSplit = 4).


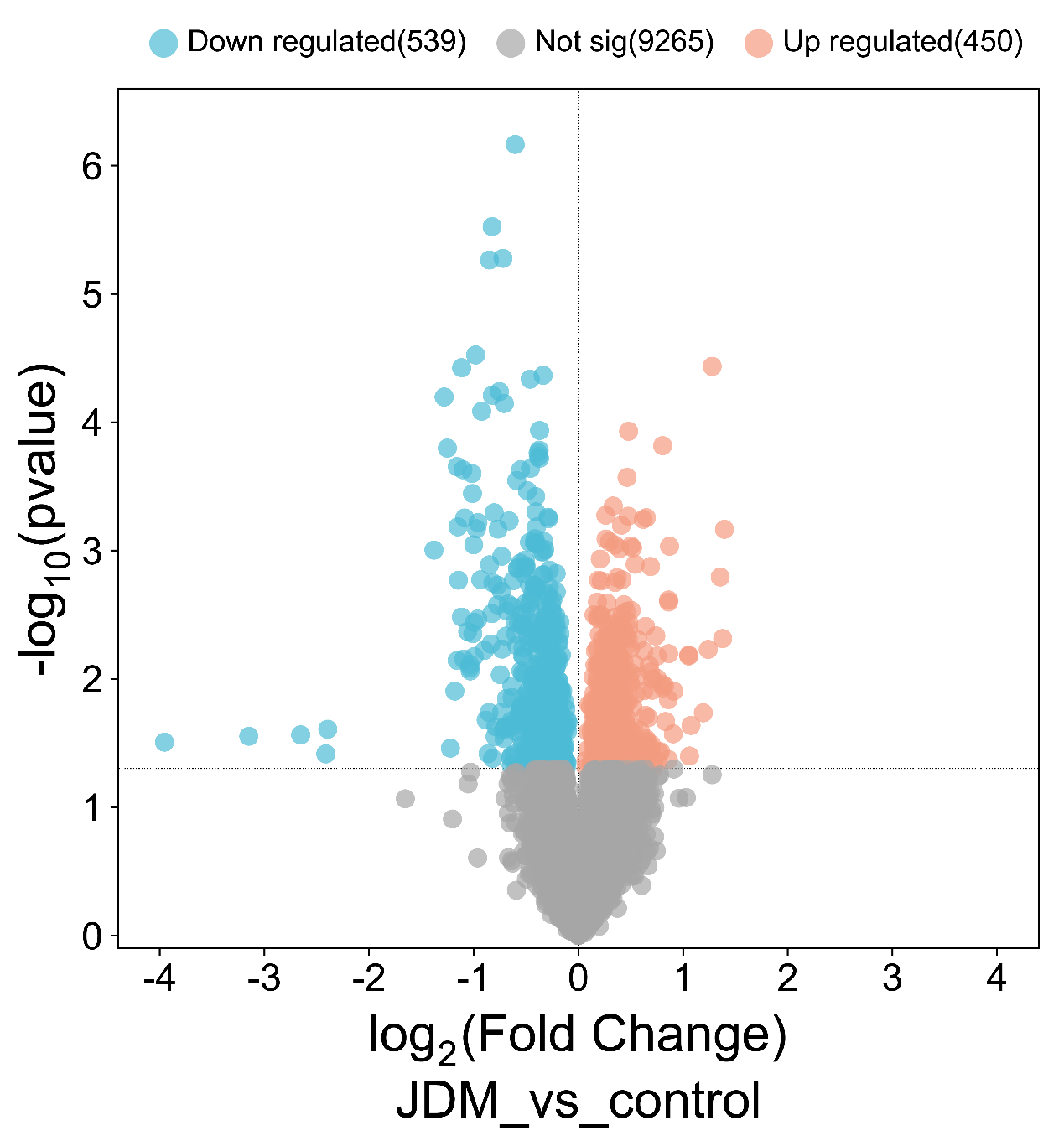


Fig. S7. Volcano map of GSE11083 differential gene analysis.


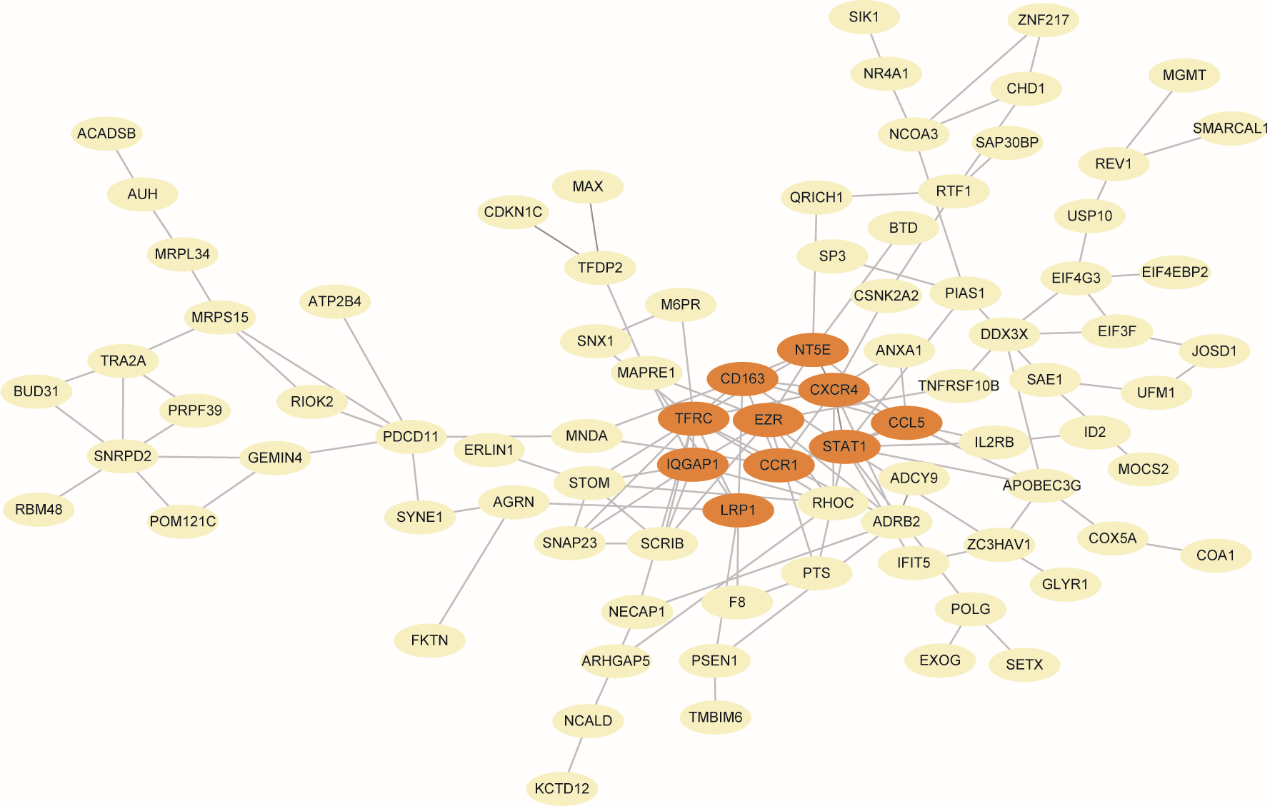


Fig. S8. Protein-protein interaction (PPI) network of the 145 genes, constructed using the STRING database. Orange nodes indicate the top 10 hub genes identified. The pale yellow nodes represent other interacting genes.
